# Motives for viewing animated sitcoms and their associations with humor styles, positivity, and self-criticism in a sample of Hungarian viewers

**DOI:** 10.1371/journal.pone.0230474

**Published:** 2020-03-17

**Authors:** Ágnes Zsila, Gábor Orosz, Zsolt Demetrovics, Róbert Urbán

**Affiliations:** 1 Institute of Psychology, ELTE Eötvös Loránd University, Budapest, Hungary; 2 Institute of Psychology, Pázmány Péter Catholic University, Budapest, Hungary; 3 Department of Psychology, Stanford University, Stanford, California, United States of America; Kyoto University, JAPAN

## Abstract

In recent years, animated situation comedies (generally known as animated sitcoms) have gained widespread popularity among young adults. Animated sitcoms often dissect sensitive social and political concerns using negative humor, exaggeration, and stereotyping. The present study aimed to explore the motives for viewing animated sitcoms using qualitative and quantitative research methods and investigate their associations with humor styles, positivity, and self-criticism in a sample of Hungarian viewers. A total of 816 Hungarian adults (54.5% female; *M*_*age*_ = 23.9 years, *SD* = 5.6) completed an online questionnaire focusing on animated sitcom viewing habits and other relevant psychological constructs. As a result, three major motive dimensions were identified: (1) social criticism, (2) fun and entertainment, and (3) relaxation. These motives were assessed by the Motives for Animated Sitcom Viewing Questionnaire (MASVQ), which demonstrated strong psychometric properties. Using a MIMIC model, multiple associations were described across motives and other psychological constructs, indicating that individuals with different levels of humor, positivity, and self-criticism are motivated to view animated sitcoms for different reasons in this sample of Hungarian viewers.

## Introduction

### Animated sitcoms

Ever since the massive success of *The Flintstones* (produced by Hanna-Barbera Productions, Inc.) in the early 1960s, prime time animated situation comedies (generally known as animated sitcoms) have gained widespread popularity among American television viewers [1]. *The Flintstones* was the first animated series in the history of American television that was not targeted solely at children [2]. Due to the unexpected success of this genre, several animated sitcoms have been produced since the late 1980s such as *The Simpsons* (created by Matt Groening in 1989), *South Park* (created by Trey Parker and Matt Stone in 1997), and *Family Guy*(created by Seth McFarlane in 1998)[1, 2]. Animated sitcoms often discuss taboo or controversial social and political topics in a US context [3], using hints of social criticism, malicious humor, strategies of exaggeration, and hyper-stereotyping in an entertaining way [4, 5]. Interest in such animated sitcoms soon expanded to an international context, resulting in that “the genre has achieved a prominent spot in contemporary Western popular culture” [5, p. 3.]. Indeed, 75% of sales related to animated sitcoms were reported from non-US markets in 1999, shortly after *South Park* and *Family Guy* were first released in the US [6].

It is particularly difficult to make estimations about the size of the global audience of animated sitcoms due to the wide variety of viewers across the episodes [3]. According to the reports by Nielsen Television Audience Measurement, *Family Guy* has attracted 6 millions of viewers a night, while *The Simpsons* gathered 4.1 million viewers, followed by *American Dad* with 4.6 million viewers, and *The Cleveland Show* with 2.6 million total viewers in 2011 among American young adults aged between 18 and 49 years [7]. In 2019, *Family Guy* still attracts 2–3 million viewers among 18–49 years old television viewers [8], while *South Park* draws 1.4 million viewers in America [9]. In Hungary, several popular animated sitcoms (e.g., *Family Guy*, *The Cleveland Show*) have been broadcasted since the late 2000s, and attracted considerable interest shortly after their first release in Hungary [10]. Indeed, three years later, *Family Guy*, *Drawn Together*, and *The Cleveland Show* were ranked at number four to six in “The 20 Most Popular Comedy Central Shows” in Hungary, while *South Park* was ranked at number 13 [11]. Despite this popularity, there is a lack of empirical research on the motives for viewing animated sitcoms. This study endeavors to fill this gap by applying both qualitative and quantitative research methods to investigate the motives of animated sitcom viewing in a sample of Hungarian animated sitcom viewers.

### Motives

Early studies in media research explored motives for viewing television [12–14]. Rubin [12] identified seven major motives: relaxation, companionship, habit, pass time, entertainment, social interaction, information, arousal, and escape. Almost two decades later, Weaver [13] examined the personality correlates of motives, and found that individuals scoring high on the neuroticism personality dimension exhibited higher motives in four aspects: pass time, companionship, relaxation, and stimulation. Potts, Dedmon and Halford [14] investigated the strength of motives, and found that the strongest motives in television viewers were entertainment, relaxation, information, and passing the time, while social [e.g., conversation topics] and escapism motives [e.g., to get away from people] were weaker motives. Besides general television motives, Gantz [15] investigated motives for viewing television sports, while another study by Perse [16] focused on local television news. The motive of passing time was identified in both studies as a major motive to view such television shows [15, 16], and the motive of entertainment was identified in relation to local television news [16] alongside some specific motives [e.g., utility].

Recent motivation research in the field of popular culture has investigated the motives relating to the use of Facebook [17], Tinder [18], Pokémon Go [19], and online games [20], for instance. The motives explored in these studies reflect some general motives identified in earlier studies with regard to television viewing (e.g., entertainment, pass time, social motives), while some specific motives were also identified (e.g., nostalgia in relation to Pokémon Go use)[17–20]. Alhabash and Ma [17] identified entertainment as the strongest motive for using Facebook, followed by information sharing, medium appeal, and escapism. Demetrovics et al. [20] identified recreation and social motives as the strongest motives for playing online games, while escape was a weaker motive. The motive of alleviating boredom was identified as a major motive in relation to Pokémon Go [19] and Tinder use [18], which is similar to the motive of passing time in earlier studies on general television viewing motives [12, 13]. This pattern suggests that the appreciation of some popular media products share some similarities with the enthusiasm towards general media content alongside specific motives, and the exploration of these motives can contribute to a better understanding of the preferences of different audiences.

The aim of this study is to contribute to the growing body of literature on motives relating to various aspects of popular culture, and identify beneficial and adverse motives in terms of psychological well-being by investigating the association of animated sitcom viewing motives with relevant psychological constructs such as humor styles, positivity, and self-criticism. The exploration of motives can provide producers and creators of such animations with a more nuanced picture of thematic and psychological aspects of a cartoon targeted at an adult audience that can attract more viewers. Indeed, the identification of main motives and individual preferences based on gender, age, and other psychological characteristics can help creators to integrate such components into their products that can allow for a more targeted effort to draw viewers’ interest towards these animations and maintain this attention in the long run. Furthermore, the exploration of motives can provide scholars with a more nuanced understanding of motive patterns underlying viewer interest in this specific media content.

### Humor styles

Provided that humor is a central element of animated sitcoms [5], humor style can be considered as an important psychological construct in the exploration of associations with animated sitcom viewing motives.

Previous research has provided evidence that psychological well-being is related to certain humor styles [21, 22]. Martin et al. [22] identified four humor styles: affiliative, self-enhancing, aggressive, and self-defeating humor. Affiliative humor is a benign form of humor that facilitates social relationships, whereas self-enhancing humor refers to the ability to maintain a humorous outlook on life, even in stressful situations. Aggressive humor is a hostile form of humor, involving the use of sarcasm, teasing, and ridicule. Self-defeating humor refers to self-disparaging humor, which is used at the expense of the self [22, 23]. According to previous studies, affiliative and self-enhancing humor styles can be considered as adaptive forms of humor, while aggressive and self-defeating humor are maladaptive styles [22, 24, 25]. Indeed, affiliative and self-enhancing humor have been associated with increased psychological well-being [23, 24] and higher positivity (i.e., dispositional optimism, hope, and happiness)[26, 27]. By contrast, aggressive humor was associated with greater hostility and aggression [22], and self-defeating humor was related to lower levels of psychological well-being [25, 28].

Martin et al. [22] found significant gender differences in humor styles, reporting an increased use of all four humor styles among males, particularly in aggressive and self-defeating humor. Furthermore, younger individuals were more likely to use affiliative and aggressive humor compared to older individuals [22]. Barbato, Graham and Perse [29] investigated the function of humor in social interactions among elders, and found that positive and expressive humor were used to express pleasure, affection, comfort, and the motive of escape, while negative humor served to gain control, seek comfort, and escape. These findings suggest that positive humor styles are used to express positive emotions, while negative humor styles may be used to alleviate frustration and negative feelings.

Although there are only a few empirical studies applying content analysis to explore humor styles represented in animated sitcoms, it is commonly known that some forms of negative humor [e.g., sarcasm and disparagement humor] are main components of such animations [4, 5]. Indeed, Eisenberg et al. [30] explored incidents of weight stigmatization in popular adolescent television programming (e.g., *Family Guy*, *The Simpsons*), and found that half of the analyzed episodes contained at least one incident of offensive weight stigmatization. In a similar vein, Pehlke et al. [31] reported that father figures are often portrayed as targets of malicious jokes in non-animated television sitcoms. Scharrer [32] found that this tendency had increased between 1950 and 1990 in family-oriented sitcoms. In relation to general humor, Weiss and Wilson [33] analyzed the five most popular prime-time family sitcoms in 1992, and found that humor was more often associated with situations that evoke negative emotions than positive emotions in the plot (e.g., angry or frightened characters were often targets of malicious humor). With regard to animated sitcoms, a recent study by Juckel, Bellman and Varan [34] analyzed humor categories and techniques in non-animated sitcoms (i.e., *The Big Bang Theory*, *Modern Family*, *The Office*) and in an animated sitcom (i.e., *Family Guy*). It was found that *Family Guy* used more derisive and offensive humor techniques such as parody, absurdity, and repulsive behavior compared to non-animated sitcoms. Besides, *Family Guy* derived humor mostly from situations discussing moral and social standards according to the findings, while non-animated sitcoms contained frequent hints of malicious and character-based humor. Juckel, Bellman and Varan [34] suggested that the genre characteristics of animated sitcoms allow for the explicit portrayal of superiority without the limitation of considering real consequences, and the portrayal of superiority is often accompanied by negative humor. Therefore, it is possible that viewers using maladaptive humor forms are more motivated to view animated sitcoms.

### Positivity

Studies investigating the association of humor styles with psychological well-being concluded that humor measures had a stronger association with assessment instruments relating to positive psychology constructs compared to measures of mental health concerns (see 22 for a review). Therefore, the present study investigates the association of motives for animated sitcom viewing with positivity to gain more insight into the relationship between motives and psychological well-being. Positivity can be defined as a tendency to view life with a generally positive outlook [35]. This construct has been associated with self-esteem, life satisfaction, and optimism [35]. According to previous findings, no gender differences could be observed in positivity; however, younger individuals expressed higher positivity and happiness compared to older individuals [35, 36].

### Self-criticism

Considering that animated sitcoms often criticize influential political or economic decisions made in a US context [3], an investigation of viewers’ attitudes towards criticism could also be relevant. In the present study, viewers’ attitudes towards criticism were approached from the perspective of self-involvement. Therefore, the construct of self-criticism was investigated, which was negatively related to happiness [37] and psychological well-being [38, 39]. Self-criticism is defined as “an exaggerated, distorted emphasis on self-definition associated with harsh personal standards” [39, p. 757]. According to Cheng and Furnham [37], females tend to report higher self-criticism, while age is unrelated to this construct. With regard to humor styles, the strongest association was found between self-defeating humor and self-criticism [39, 40], while affiliative and aggressive humor were weakly associated with self-criticism [39].

Overall, these psychological constructs (i.e., humor styles, positivity, and self-criticism) could be considered as relevant constructs in the investigation of animated sitcom viewing motives with psychological dispositions.

### The aim of the study

This study endeavors to contribute to the literature on motives linked to the use and consumption of popular culture media products, and identify potentially adaptive and maladaptive motives in relation to animated sitcom viewing by investigating the association of Hungarian viewers’ motives with various aspects of psychological well-being that could be relevant to the present research based on the literature. To attain these goals, two aims have been formulated: 1) to explore motives for animated sitcom viewing in a sample of Hungarian viewers, using qualitative and quantitative research methods, and construct an assessment instrument that assesses these motives, 2) to investigate the association of motives with relevant psychological constructs such as humor styles, positivity, and self-criticism. With regard to the sample characteristics, it should be noted that the present study was conducted on a sample of Hungarian adult viewers who must have been dedicated fans of this genre, considering their active membership in various discussion forums for cartoons. Their critical expertise on animated sitcoms may result in some biases in the perception of humor, aggression, or positivity with regard to these cartoons or themselves, which should be taken into account when considering the present findings. It should also be noted that this sample of Hungarian adult viewers is not representative of the general audience of television viewers.

## Materials and methods

The study was carried out with the approval of the Institutional Review Board of ELTE Eötvös Loránd University and was in accordance with the Declaration of Helsinki. Approval number: 2018/14-2. Written consent was obtained.

### Participants and procedure

Participants were recruited using an online questionnaire that was advertised in four Hungarian online communities devoted to cartoons that had more than 2,000 members (Hungarian Cartoon Fans, Cartoons for Everyone, Best Old Cartoons, Cartoons Online Facebook groups). Data was gathered during the spring of 2017. Before completing the questionnaire, participants were requested to provide informed consent by ticking a box if they were over 18 years of age and agreed to the terms. Participation in the survey was voluntary and anonymous. The study was carried out with the approval of the Institutional Review Board of the research team’s university and was in accordance with the Declaration of Helsinki.

A total of 816 adult animated sitcom viewers (54.5% female; *M*_*age*_ = 23.9 years, *SD* = 5.6) participated in the survey. The majority of participants reported having a high school degree (59.3%) as the highest level of education, whereas 24.8% had post-secondary degree, and only 15.9% had primary school degree or lower. Approximately one-third of participants studied at the time of the data collection (34.4%), while 31.3% had a full-time or part-time job, and 28.8% studied and worked. Only a minority of participants neither studied, nor worked (5.5%).

### Item construction to assess motives for viewing animated sitcoms

To explore the motives underlying animated sitcom viewing, a qualitative data collection was carried out on a sample of 62 participants (67.7% female; *M*_*age*_ = 24.0 years, *SD* = 3.6) who reported being regular animated sitcom viewers. Participants were recruited from the same four Hungarian online communities devoted to cartoons (having more than 2,000 members) as for the subsequent quantitative data collection. Survey participation was voluntary. Using an online questionnaire, participants over 18 years of age were invited to complete the following sentence: “I view animated sitcoms because…” A total of 191 responses were recorded, of which 186 were considered for further analysis (the content of five responses were not related to motives). Subsequently, three expert raters with former experience in motivation research and qualitative data analysis independently categorized participants’ responses. In the case of three responses, consensus was achieved after raters were allowed to discuss the categorization of the respective responses. As a result of the content analysis, 104 responses (55.9%) were categorized into four major motive dimensions: Social Criticism (*n* = 43; 23.1%), Fun (*n* = 33; 17.8%), Entertainment (*n* = 14; 7.5%), and Relaxation (*n* = 14; 7.5%). Furthermore, nine responses (4.8%) reflected more than one category of the above mentioned categories, including Fun (*n* = 8; 4.3%), Social Criticism (*n* = 6; 3.2%), Entertainment (*n* = 4; 2.2%), and Relaxation (*n* = 1; 0.5%).

The *Social Criticism* dimension refers to the motive of viewing television programs that criticize society in a malicious and sarcastic way. The dimension of *Fun* refers to the motive of viewing a funny television program that makes viewers laugh without hints of malicious humor. The motive of *Entertainment* represents responses reflecting that viewers derive amusement and pleasure from this type of animation. *Relaxation* refers to the motive of recreation.

The motives of Entertainment and Relaxation were also identified by Rubin [12] in relation to television viewing motives. Therefore, all six items (three items per subscale) from the Relaxation and Entertainment subscales were derived from the television viewing motives scale [12] in the present item construction due to the considerable overlap between the items’ content and the responses collected in the present qualitative survey. Moreover, two items were added to each subscale reflecting other aspects of entertainment (item 14 and 15 in Table 2) and relaxation (item 19 and 20 in Table 2) that needed to be considered based on the qualitative survey. With regard to the Social Criticism and Fun motive dimensions, following the exclusion of duplicates, five items remained that were rephrased in order to reflect the conceptual content of the motive dimensions in a wide variety of different ways. Therefore, each subscale (Social Criticism, Fun, Entertainment, and Relaxation) comprised five items.

Beside the four major motive dimensions, a small proportion of responses in the qualitative survey reflected motives of viewing animated sitcoms in order to avoid being bored when there is nothing better to do (*n* = 6; 3.2%). In addition, a small minority of the respondents reported viewing animated sitcoms because these cartoons are short (*n* = 5; 2.7%), interesting (*n* = 5; 2.7%), up-to-date (*n* = 4; 2.2%), and have depth (*n* = 6; 3.2%). However, none of these statements could offer sufficient information for the raters to constitute new motive dimensions. Other responses (25.3%) were unique statements of which separate categories could not be established.

As a result of the item construction process, a 20-item version of the Motives for Viewing Animated Sitcom Questionnaire (MVASQ) was considered as a basis for further scale development (see all items in Table 2). In the follow-up quantitative research, participants were asked to indicate the extent to which each statement was characteristic of them using a five-point Likert-scale (ranging from 1 = ‘very uncharacteristic of me’ to 5 = ‘very characteristic of me’). The instruction, adapted from the study by Rubin [12], was as follows: “People view animated sitcoms for different reasons. Some reasons are listed below. Please indicate to what extent each of the following statements is characteristic of you by clicking on the appropriate response: 1 –very uncharacteristic of me, 2 –slightly characteristic of me, 3 –moderately characteristic of me, 4 –characteristic of me, 5 –very characteristic of me. There is no wrong or right answer. We are only interested in your motives for viewing animated sitcoms.”

### Measures

Data regarding major demographics (i.e., gender, age, education, current studies and/or work experience) were collected. Beside demographics, several relevant psychological constructs were assessed (listed below).Items of the assessment instruments were translated and back-translated following the protocol by Beaton, Bombardier [41].

#### Animated sitcom viewing

Participants were asked to indicate how often they had viewed animated sitcoms during the past year (1 = 1–5 times per year, 2 = 6–12 times per year, 3 = 1–4 times per month, 4 = 1–2 times per week, 5 = 3–5 times per week, 6 = daily or almost daily). In addition, participants were requested to name those animated sitcoms they had viewed during the past year.

#### Humor styles

Participants’ humor styles were assessed using the 32-item Humor Styles Questionnaire [HSQ; 22]. The HSQ assesses four different humor styles on four subscales: Affiliative humor (e.g., “I laugh and joke a lot with my closest friends.”) (α = 0.79), Self-Enhancing humor (e.g., “If I am feeling depressed, I can usually cheer myself up with humor.”) (α = 0.78), Aggressive humor (e.g., “If someone makes a mistake, I will often tease them about it.”) (α = 0.71), and Self-Defeating humor (e.g., “I let people laugh at me or make fun at my expense more than I should.”) (α = 0.70). All four subscales comprise eight items. According to previous research, affiliative and self-enhancing humor should be considered as positive humor styles, while aggressive and self-defeating humor as negative humor styles [42]. Participants were asked to indicate the extent of which each statement was characteristic of them using a seven-point Likert scale (1 = ‘totally disagree’, 7 = ‘totally agree’).

#### Positivity

Participants’ positive orientation towards self, life, and the future was assessed using the eight-item Positivity Scale [P Scale; 35]. A typical item of this one-dimensional scale states: “I have great faith in the future”. Participants indicated their level of agreement with the statements on a five-point Likert-scale (ranging from 1 = ‘strongly disagree’ to 5 = ‘strongly agree’). Cronbach’s alpha was 0.77 in the present study.

#### Self-criticism

Participants’ self-criticism was assessed using the Self-Critical subscale of the Theoretical Depressive Experiences Questionnaire [TDEQ; 43]. The Self-Critical subscale comprises seven items (e.g., “I tend to be very critical of myself.”). Participants indicated their level of agreement with each statement on a seven-point Likert-scale (ranging from 1 = ‘strongly disagree’ to 7 = ‘strongly agree’). Cronbach’s alpha was 0.86 in the present study.

### Statistical analysis

Descriptive statistics were calculated using IBM SPSS version 22.0 (IBM SPSS Inc., Chicago, Illinois).The data set was screened for duplicates using the ‘Identify duplicate cases’ option in SPSS. As a result, no matching records were found with regard to study-relevant variables. To investigate the psychometric properties of the factor structure of the MVASQ, exploratory factor analysis (EFA) and confirmatory factor analysis (CFA) were conducted using Mplus 6.1 [44]. The EFA was carried out using an oblique (geomin) rotation, whereas CFAs were carried out applying maximum likelihood estimation with robust standard errors (MLR). The first CFA, which was conducted on the total sample (*N* = 816), yielded inadequate fit indices. Therefore, an EFA and another CFA were performed on two different samples (*n* = 408 for both analyses) after randomly half-splitting the total sample. The following fit indices were used to evaluate the goodness of fit of the models [45, 46]: the Comparative Fit Index (CFI: ≥ 0.95 for good, ≥ 0.90 for acceptable), the Tucker-Lewis index (TLI: ≥ 0.95 for good, ≥ 0.90 for acceptable), and the Root-Mean-Square Error of Approximation (RMSEA: ≤ 0.06 for good, ≤ 0.08 for acceptable), with its 90% confidence interval, and the Standardized Root Mean Square Residual (SRMR:≤ 0.05 for good, ≤ 0.10 for acceptable). Internal consistency (Cronbach’s alpha) was considered as acceptable above values of 0.60 [47].

In the final step, a multiple indicators multiple causes (MIMIC) confirmatory factor analysis was conducted on the final structural model of the MVASQ to investigate the associations of animated sitcom viewing motives with major demographics and other relevant variables. MIMIC models allow for the estimation of the effect of indicators on latent variables while controlling for the direct effect of other variables on latent constructs. In the present study, the MIMIC model comprises the motives for animated sitcom viewing as latent variables and the respective items as indicators of latent constructs. This part of the constructed model is equivalent to a CFA. However, the model also incorporates exogenous variables (i.e., gender, age, humor styles, self-criticism, positivity) in the present study, which allows for the investigation of associations between animated sitcom viewing motives and other relevant variables such as humor styles, self-criticism, and positivity, and makes it possible to explore motivational differences across gender and age.

### Ethics

The study was carried out with the approval of the Institutional Review Board of the research team’s university and was in accordance with the Declaration of Helsinki. Participants were informed about the study’s aim, and provided written consent before completing the questionnaire.

## Results

### Descriptive statistics

Descriptive statistics relating to animated sitcom viewing and other study-relevant variables are presented in Table 1.

**Table 1:** Descriptive statistics of animated sitcom viewing, humor styles, self-criticism, and positivity in the total sample of animated sitcom viewers.

	Participants (*N* = 816)
**Animated sitcom viewing**	
Animated sitcoms viewed in the past year *n* (%)*	
Family Guy	670 (82.1%)
South Park	485 (59.4%)
The Simpsons	274 (33.6%)
American Dad	116 (14.2%)
Brickleberry	115 (14.1%)
Frequency of viewing *n* (%)	
Daily or almost daily	186 (22.8%)
3–5 times per week	163 (20.0%)
1–2 times per week	177 (21.7%)
1–4 times per month	195 (23.9%)
6–12 times per year	55 (6.7%)
1–5 times per year	40 (4.9%)
**Humor styles**(range: 1–7) *Mean(SD)*	
Affiliative humor	5.69 (0.15)
Self-enhancing humor	4.85 (1.15)
Aggressive humor	3.98 (1.09)
Self-defeating humor	4.05 (1.08)
**Self-criticism**(range: 1–7) *Mean(SD)*	4.77 (1.39)
**Positivity**(range: 1–5) *Mean(SD)*	3.25 (0.78)

* Only the five most frequently reported animated sitcoms are presented in this table.

The most frequently mentioned animated sitcoms that had been viewed by participants in the past year were *Family Guy* (82.1%) and *South Park* (59.4%), followed by *The Simpsons* (33.6%), *American Dad* (14.2%), and *Brickleberry* (14.1%). A small proportion of participants also reported viewing *Archer* (11.9%). Other animated sitcoms mentioned by some viewers were *The Cleveland Show* (7.0%), *Rick and Morty* (5.0%), *Futurama* (4.9%), and *Bob’s Burgers* (4.4%). More than half of the participants (62.2%) reported viewing animated sitcoms at least once a week, whereas 23.9% viewed animated sitcoms only 1–4 times a month, and only a small proportion of participants (11.6%) viewed these cartoons a few times in the past year, which suggests that the majority of participants were dedicated viewers of American animated sitcoms.

### Exploratory (EFA) and confirmatory (CFA) factor analysis

Confirmatory factor analysis (CFA) was conducted on the total sample (*N* = 816) in order to test the psychometric properties of the MVASQ that was constructed based on previous qualitative research. The four-factor, 20-item MVASQ failed to have an acceptable model fit, since the value of the SRMR exceeded the cutoff score of 0.10 for acceptance (*χ*^*2*^ = 875.80; *df* = 166; *p*< 0.001; CFI = 0.926; TLI = 0.916; RMSEA = 0.073 [0.068–0.077], SRMR = 0.233). Furthermore, inter-factor correlations were particularly high (between 0.60 and 0.95; mean *r* = 0.77). Therefore, an exploratory factor analysis was needed to investigate the factor structure of the MVASQ. For this purpose, the total sample was randomly half-split for the EFA (*n* = 408) and the subsequent CFA (*n* = 408).

**Table 2:** Factor loadings of all items of the preliminary item pool of the motives for viewing animated sitcoms (MVASQ) based on the exploratory factor analysis (EFA) (*n* = 408).

Item	Factor 1: Social Criticism	Factor 2: Fun and Entertainment	Factor 3: Relaxation
1. … it’s a good kind of social criticism	**0.717**	0.064	0.085
2. … it’s a reflection of self-ridicule of people in society	**0.823**	0.013	0.032
3. …it breaks social taboos	**0.766**	–0.023	0.113
4. … it’s scandalous	**0.560**	0.009	–0.014
5. …it’s malicious and sarcastic	0.529	0.362	–0.041
6. … it’s funny	0.000	**0.859**	–0.012
7. … it’s humorous	–0.022	**0.895**	0.034
8. … I laugh hard when I watch it	–0.010	**0.900**	–0.009
9. … I like these jokes	0.078	**0.771**	0.058
10. … it makes me laugh	0.007	**0.945**	–0.067
11. … it entertains me*	0.034	**0.692**	0.176
12. …it’s enjoyable*	0.039	0.503	0.372
13. … it amuses me*	–0.009	**0.716**	0.220
14. … I enjoy watching it	–0.012	0.551	0.326
15. … it’s refreshing	0.159	0.237	0.473
16. … it relaxes me*	0.177	–0.012	**0.655**
17. … it allows me to unwind*	–0.010	0.024	**0.728**
18. … it’s a pleasant rest*	–0.085	0.387	0.589
19. … it chills me out	0.126	–0.013	**0.680**
20. … switches off my brain	0.104	0.032	**0.626**

Factor loadings for the EFA was calculated using an independent sample of 408 participants after randomly half-splitting the total sample (*N* = 816).

* Items marked with an asterisk are derived from the television viewing motives scale by Rubin (1983).

Bold-faced factor loadings belong to items that constitute the final version of the MVASQ.

An exploratory factor analysis was conducted on a sample of 408 participants (52.7% female; *M*_*age*_ = 24.2 years, *SD* = 6.0) (see Table 2 for details). The eigenvalues of the first five factors were 10.47, 1.97, 1.46, 0.75, and 0.65. Beside the eigenvalues, the three-factor model demonstrated an acceptable model fit (*χ*^*2*^ = 343.27; *df* = 133; *p*< 0.001; CFI = 0.958; TLI = 0.939; RMSEA = 0.063 [0.054–0.071], SRMR = 0.025). Therefore, this model was considered as a basis for further investigation.

According to the EFA, items of the respective factors were retained based on the following criteria: (i) if the factor loading of an item was at least 0.5; (ii) if there were no high cross-loadings (i.e., when multiplying a factor loading twice and the value has not exceeded the highest factor loading on another factor); (iii) if the factor had at least two items following the first two steps of item selection.

Based on this procedure, five items were deleted due to high cross-loadings (i.e., item 5, 12, 14, 15, 18 in Table 2), which resulted in a 15-item three-factor model: Social Criticism (four items), Fun and Entertainment (seven items; five of the Fun factor and two of the Entertainment factor), and Relaxation (four items). According to the subsequent confirmatory factor analysis on another independent sample of 408 participants (54.7% female; *M*_*age*_ = 24.1 years, *SD* = 5.6), this model had a good fit to the data (*χ*^*2*^ = 202.70; *df* = 88; *p*< 0.001; CFI = 0.968; TLI = 0.962; RMSEA = 0.057 [0.047–0.067], SRMR = 0.061). Factor loading were between 0.54 and 0.93 (p < 0.001), and the mean of the factor loadings were 0.74 for the Social Criticism, 0.89 for the Fun and Entertainment, and 0.68 for the Relaxation motive dimension (see Table 3 for details).

**Table 3:** Items, descriptive statistics, and reliability indices of the motives for viewing animated sitcoms questionnaire (MVASQ).

I view animated sitcoms because…	α	Descriptive statistics			Factor loadings
		Range	Mean	SD	CFA
Social Criticism	0.83	1–5	3.36	1.03	
1. … it’s a good kind of social criticism					0.79
2. … it’s a reflection of self-ridicule of people in society					0.81
3. …it breaks social taboos					0.83
4. … it’s scandalous					0.54
Fun and Entertainment	0.95	1–5	4.19	0.81	
5. … it’s funny					0.89
6. … it’s humorous					0.93
7. … I laugh hard when I watch it					0.90
8. … I like these jokes					0.86
9. … it makes me laugh					0.91
10. … it entertains me*					0.85
11. … it amuses me*					0.87
Relaxation	0.81	1–5	3.34	0.93	
12. … it relaxes me*					0.66
13. … it allows me to unwind*					0.73
14. … it chills me out					0.68
15. … switches off my brain					0.63

α = Cronbach’s alpha; SD = standard deviation; CFA = confirmatory factor analysis.

Factor loadings for the CFA were calculated using an independent sample of 408 participants after randomly half-splitting the total sample (*N* = 816). Descriptive statistics were calculated using the total sample.

The instruction, which is a modified version of the instruction for the television viewing motives scale constructed by Rubin [12], is as follows: People view animated sitcoms for different reasons. Some reasons are listed below. Please indicate to what extent each of the following statements is characteristic of you by clicking on the appropriate response: 1 –very uncharacteristic of me, 2 –slightly characteristic of me, 3 –moderately characteristic of me, 4 –characteristic of me, 5 –very characteristic of me. There is no wrong or right answer. We are only interested in your motives for viewing animated sitcoms.

* Items marked with an asterisk are derived from the television viewing motives scale by Rubin [12]

Cronbach’s alphas of the subscales were above 0.80, indicating a high internal consistency. With regard to inter-factor correlations, the motive of Social Criticism was positively associated with both Fun and Entertainment (*r* = 0.47; *p*< 0.001) and Relaxation (*r* = 0.47; *p*< 0.001). The strongest association could be observed between the motives of Fun and Entertainment and Relaxation (*r* = 0.68; p < 0.001).

Considering the strong psychometric properties of the three-factor model, this structure was retained and used in further data analysis.

### Multiple indicators multiple causes model (MIMIC) on animated sitcom viewing motives, major demographics, and other relevant variables

To investigate the associations of motives for animated sitcom viewing with demographics and other relevant variables, eight exogenous observed variables (i.e., gender, age, the four humor styles, self-criticism, and positivity) were added to the CFA model. This complemented model demonstrated a good fit to the data (*χ*^*2*^ = 538.44; *df* = 183; *p*< 0.001; CFI = 0.953; TLI = 0.943; RMSEA = 0.049 [0.044–0.054], SRMR = 0.028). Associations between the components of this model are presented in Table 4. All correlations between the study variables are presented in the Appendix.

**Table 4:** A multiple indicators multiple causes model (MIMIC) representing the associations of motives for animated sitcom viewing with major demographics, humor styles, self-criticism, and positivity (*N* = 806).

Explanatory variables	Outcome variables Animated sitcom viewing motives *beta (SE)*		
	Social Criticism	Fun and Entertainment	Relaxation
Gender	–0.04 (0.04)	–0.12 (0.03)**	–0.02 (0.04)
Age	0.03 (0.05)	–0.02 (0.04)	0.00 (0.04)
Affiliative humor	0.00 (0.04)	0.13 (0.04)**	–0.01 (0.04)
Self-enhancing humor	0.28 (0.05)***	0.20 (0.05)***	0.18 (0.05)***
Aggressive humor	0.14 (0.04)***	0.11 (0.04)**	0.03 (0.04)
Self-defeating humor	–0.02 (0.05)	–0.04 (0.04)	0.01 (0.05)
Positivity	0.11 (0.05)*	0.09 (0.05)	0.10 (0.05)*
Self-criticism	0.17 (0.05)***	0.14 (0.05)**	0.22 (0.05)***
R^2^	12.8%	11.9%	6.8%

*** *p*< 0.001

** *p*< 0.01

* *p*< 0.05.

*beta (SE) =* standardized coefficient and its standard error

Gender is coded as 0 = male, 1 = female.

Age, humor styles, self-criticism, and positivity are continuous variables.

With regard to demographic characteristics, males scored higher than females on the Fun and Entertainment motive dimension (*β* = –0.12; *p* = 0.001), whereas age was not associated with animated sitcom viewing motives.

The motive of Social Criticism was associated with a positive (i.e., self-enhancing humor) (*β* = 0.28; *p*< 0.001) and a negative humor style (i.e., aggressive humor) (*β* = 0.14; *p*< 0.001). Fun and Entertainment demonstrated positive association with two positive humor styles (*β* = 0.20; *p*< 0.001 for Self-enhancing humor and *β* = 0.13; *p* = 0.003 for Affiliative humor) and a negative humor style (*β* = 0.11; *p* = 0.003 for Aggressive humor). Relaxation was associated only with a positive humor style (*β* = 0.18; *p*< 0.001 for Self-enhancing humor). However, these associations were generally weak. Motives yielded the strongest associations with Self-enhancing humor style. Self-defeating humor was unrelated to either animated sitcom viewing motive.

All three motives were positively associated with self-criticism. Furthermore, Social Criticism (*β* = 0.11; *p* = 0.02) and Relaxation (*β* = 0.10; *p* = 0.04) were related to positivity, while Fun and Entertainment was not associated with positivity (*β* = 0.09; *p* = 0.10). These associations were again weak.

## Discussion

### Definition and assessment of motives for animated sitcom viewing

According to the present qualitative investigation, four major higher motives were identified such as Social Criticism, Fun, Entertainment, and Relaxation; however, as a result of a series of factor analyses, motive factors were reduced to three dimensions such as Social Criticism, Fun and Entertainment, and Relaxation. Social criticism refers to the motive of viewing media content that breaks social taboos and ridicule people in society. Fun and Entertainment refers to the motive of viewing animated sitcoms for the jokes, laughs, and the amusement that can be derived from this kind of animation. Finally, relaxation refers to the motive of spending time to rest while watching animation. These motives were considered as a basis for the construction of the Motives for Animated Sitcom Viewing Questionnaire (MASVQ). The MASVQ yielded strong psychometric properties in terms of factor structure and internal consistency. Therefore, the MASVQ can be considered as an appropriate assessment tool to measure motives for animated sitcom viewing.

The motive of Fun and Entertainment and Relaxation share considerable similarities with the motive factors of Entertainment and Relaxation reported in early studies that investigated motives for general television viewing [12–14]. Furthermore, entertainment was also identified as a main motive for viewing local television news [16] and using Facebook [17]. This pattern reflects that the majority of motives relating to animated sitcom viewing share common features with those identified in previous studies in relation to general television viewing. In addition, one specific motive was described (i.e., Social criticism) that has not been identified in previous research, suggesting that the portrayal of social criticism in animation is a specific aspect of this media genre that appeals to a broad audience.

### The association of motives with humor styles, positivity, and self-criticism

The associations of motives with relevant psychological constructs were also explored in this study. Provided that substantial gender and age effects have been reported in previous studies concerning humor styles, positivity, and self-criticism [22, 35, 37], and considering the fact that these personal dispositions are associated constructs [22, 38, 39], a multiple indicators multiple causes (MIMIC) model was applied which allowed for the estimation of the effect of indicators on latent constructs while controlling for all other variables included in the model.

With regard to demographic characteristics, males were more likely to view animated sitcoms for fun and entertainment than females. This result is in line with previous findings indicating that males have a higher tendency to engage in all forms of humor compared to females [22]. In turn, no association could be found between motives and age. This result suggests that both younger and older viewers can be motivated to seek media content that criticizes people in society, derive amusement from such animation, and have a pleasant rest while watching it.

In relation to humor styles, affiliative humor was positively associated with the motive of fun and entertainment, which suggest that viewers with an increased use of affiliative humor may benefit more from the amusement that can be derived from this media content since these viewers tend to share their experience with humorous situations, which in turn can enhance interpersonal relationships. This process can possibly contribute to the formation of fan communities dedicated to enthusiasts of animated sitcoms.

Self-enhancing humor was related to all three motive dimensions, which suggests that viewers with an increased use of self-enhancing humor may be more motivated to view animated sitcoms in general. Animated sitcoms that portray the incongruities and absurdities of life in a humorous form may facilitate the maintenance of a general positive outlook on life even in times of stress. Besides, Weiss and Wilson [33] suggested that negative humor in sitcoms can possibly enhance adaptive coping with negative life events in young individuals, since they learn how to use humor in such situations instead of using maladaptive coping strategies or emotional reactions (e.g., escape, feeling embarrassed or frightened). It is possible that animated sitcoms also encourage viewers to use humor in negative emotional situations.

By contrast, aggressive humor was related to the motives of fun and entertainment and social criticism but unrelated to the motive of relaxation. This result suggests that viewers with a tendency to engage in aggressive humor are more likely to become attracted to animated sitcoms for the criticism and the amusement that can be derived from its humorous portrayal. This result is in line with previous studies suggesting that viewers who appreciate negative humor are more likely to have a preference for television shows that portray stigmatization, offensive behavior, and superiority using malicious hints of humor [30, 34].

Self-defeating humor was not associated with motives, which indicates that individuals with a tendency to use self-defeating humor are not motivated to view animated sitcoms for relaxation, fun and entertainment, or the portrayal of social criticism. In connection with this result, it was found that individuals with a higher level of self-criticism were more motivated to view animated sitcoms for all these reasons than individuals with a generally lower level of self-criticism. One possible explanation for this result may be that individuals with a high level of self-criticism consciously avoid using self-defeating humor in order to maintain their self-esteem; therefore, individuals with this humor style are not motivated to view animations that entertain viewers by using strategies of malicious humor and criticism. However, results also indicate that those with higher levels of self-criticism appreciate the humorous portrayal of social criticism in animated sitcoms. This result is in line with the findings reported by Frewen et al. [40], who considered perfectionism and self-criticism as closely associated constructs in their investigation, suggesting that highly self-critical individuals may also be critical of others.

Positivity was only weakly associated with two motives: social criticism and relaxation, while the motive of fun and entertainment failed to reach the level of significance. This result indicates that individuals with a generally positive outlook on life are slightly more motivated to view animated sitcoms in general than individuals with a less positive outlook on life. It is possible that the irrationalities of life portrayed in animated sitcoms facilitate the maintenance of a positive perspective in viewers with high levels of happiness, optimism, and life satisfaction. Besides, it is also possible that the absurdities of life portrayed in animated sitcoms encourage viewers to use humor in negative or challenging real-life situations to alleviate negative feelings associated with the situation, as was suggested by Weiss and Wilson [33].

### Limitations

This study has several limitations that need to be discussed. First, the present findings may not be generalizable to the whole population of Hungarian animated sitcom viewers due to the convenience sampling method. In addition, the present investigation exclusively focused on Hungarian viewers, which limits the generalizability of the findings to viewers of other cultures. Future research should extend this investigation to a broader audience in order to gain a more nuanced picture of viewer motives. For instance, some social and political concerns dissected in animated sitcoms are relevant in a US context; therefore, animated sitcoms may have a different meaning for American and non-American viewers, which can possibly be reflected in a different motivational background. Second, only the most frequently reported motives were considered as a basis of the assessment of motives in the present study which allowed for the construction of a separate motive factor. However, some relevant motives may be ignored in the quantitative data analysis due to the lack of information provided by respondents in the qualitative survey. Future research should investigate these motives in more depth. Third, this study used a cross-sectional research design, which limits the possibility of drawing clear conclusions about the direction of associations. Further research is needed to develop a more nuanced understanding of the causal mechanisms underlying the associations of animated sitcom viewing motives with humor styles, positivity, and self-criticism. Finally, this study relied on self-report measures; therefore, various biases may affect the results (e.g., social desirability, inaccurate memory). Future research should also investigate the associations of motives with other psychological constructs (e.g., personality traits, social relationships) in order to clarify which animated sitcom viewing motives can be considered as adaptive and maladaptive motives in terms of psychological well-being. Results of the present study pointed out the complexity of these motives, preventing a clear classification of positive and negative motive dimensions. Future research should also investigate possible similarities and differences between animated and non-animated sitcoms with regard to viewer motives. The present study exclusively focused on the motives for viewing animated sitcoms; therefore, the results are not generalizable to non-animated sitcoms. A recent study by Juckel, Bellman and Varan [34] has pointed out some important differences between animated and non-animated sitcoms in humor techniques. Drawing on this finding, it is possible that there are major differences in viewer motives for animated and non-animated sitcoms, considering that some specific humor techniques portrayed in sitcoms may appeal to viewers with certain personality characteristics, preferences, and motivational background.

## Conclusion

Despite the limitations, the present investigation has some implications. This study was among the first to investigate animated sitcom viewing motives employing both qualitative and quantitative research methods, using a sample of Hungarian adults. As a result, three major motives were identified (i.e., Social Criticism, Fun and Entertainment, and Relaxation), which can be assessed by the Motives for Animated Sitcom Viewing Questionnaire (MASVQ). The MASVQ demonstrated strong psychometric properties in this study. According to the results, it can also be concluded that individuals with different humor styles and different levels of positivity and self-criticism are motivated to view animated sitcoms for different reasons in this sample of Hungarian adults. A cross-cultural research would allow for the identification of general and culture-specific motives, and extend knowledge on the psychological characteristics associated with animated sitcom viewing motives.

Overall, the findings reported in this study may provide creators of animated sitcoms with a more detailed picture of the content and psychological characteristics of a cartoon that can attract considerable viewer interest among adults. These findings may also allow producers to create more targeted content to maintain viewers’ interest in their animations in the long run. Finally, these results point out some elements (e.g., fun and entertainment) of animated sitcoms that appeal more strongly to some specific audiences (e.g., male viewers) than other aspects. The incorporation of such elements into the content of animated sitcoms targeted at specific audiences may increase the efficacy of efforts invested in enhancing viewer interest and dedication. In addition, these findings could possibly provide scholars with a more nuanced picture of motives underlying viewer interest in this specific media content, and offer more insight into some relevant motives for future research that aims to clarify the similarities and differences between motives for viewing animated and non-animated situation comedies.

## Supporting information

S1 Appendix Estimated bivariate correlations between motives for animated sitcom viewing and major demographics, humor styles, positivity, and self-criticism. (DOCX)
