# Supplementary material for: Motives for viewing animated sitcoms and their associations with humor styles, positivity, and self-criticism in a sample of Hungarian viewers
Source: PLoS One. 2020 Mar 17;15(3):e0230474. doi: 10.1371/journal.pone.0230474 (PMC7077815; doi:10.1371/journal.pone.0230474)
Supplement: S1 Appendix — (DOCX) [file pone.0230474.s001.docx]

**Appendix**

**Appendix: Estimated bivariate correlations between motives for animated sitcom viewing and major demographics, humor styles, positivity, and self-criticism**

|  | 1. | 2. | 3. | 4. | 5. | 6. | 7. | 8. | 9. | 10. |
| --- | --- | --- | --- | --- | --- | --- | --- | --- | --- | --- |
| 1. SocialCriticism | – |  |  |  |  |  |  |  |  |  |
| 2.Fun and Entertainment | **0.50** | – |  |  |  |  |  |  |  |  |
| 3. Relaxation | **0.45** | **0.66** | – |  |  |  |  |  |  |  |
| 4. Age | 0.03 | –0.01 | –0.01 | – |  |  |  |  |  |  |
| 5. Gender | –0.03 | –**0.11** | 0.01 | 0.03 | – |  |  |  |  |  |
| 6. Affiliative humor | **0.15** | **0.23** | 0.07 | 0.03 | –0.01 | – |  |  |  |  |
| 7. Self-enhancing humor | **0.30** | **0.25** | **0.18** | **0.10** | **0.08** | **0.43** | – |  |  |  |
| 8. Aggressive humor | **0.17** | **0.17** | 0.05 | –**0.12** | –**0.15** | **0.20** | **0.15** | – |  |  |
| 9. Self-defeating humor | **0.13** | **0.10** | **0.12** | –**0.15** | –0.01 | **0.21** | **0.32** | **0.24** | – |  |
| 10. Positivity | **0.12** | **0.13** | 0.05 | **0.10** | –0.01 | **0.32** | **0.34** | 0.02 | –**0.10** | – |
| 11. Self-criticism | 0.04 | 0.01 | **0.13** | –**0.14** | **0.09** | **-0.20** | **-0.17** | -0.06 | **0.26** | –**0.53** |

*Note*.*N*=806. Boldfacedcorrelations are significantatleast*p*<0.05.
